# Exploring female fitness app users’ motivations and perceptions: A qualitative study

**DOI:** 10.1097/MD.0000000000040076

**Published:** 2024-11-01

**Authors:** Le Lyu, Nor Eeza Zainal Abidin, Hutkemri Zulnaidi

**Affiliations:** a Faculty of Sports and Exercise Science, Universiti Malaya, Kuala Lumpur, Malaysia; b Department of Mathematics and Science Education, Faculty of Education, Universiti Malaya, Kuala Lumpur, Malaysia; c Faculty of Sports and Exercise Science, Universiti Malaya, Kuala Lumpur, Malaysia.

**Keywords:** continuance intention, female users, fitness apps, value

## Abstract

**Background:**

Women make up nearly 60% of fitness App users and play an important role in the operation and development of these Apps. Despite the widespread adoption of fitness Apps in recent years due to their many physical and mental benefits, the use of Apps tends to be short-term, and user engagement is relatively low. Little is known about the factors that motivate female users to consistently use fitness Apps. This study aimed to study attributes of fitness Apps that are most likely to influence female users during their use of Apps.

**Methods:**

The means-ends chain theory and the soft-laddering interview technique were used to analyze the attributes that influenced females’ perceptions of fitness apps, focusing on the features most likely to provide them with benefits and value.

**Result:**

The results of the study indicated that instructiveness, interaction, personalization, ease of use, and convenience were the most important attributes valued by female fitness App users; peer support, entertainment, and exercise adherence were the most important consequences; and health, self-fulfillment, enjoyment of life and sense of achievement were the most important values.

**Conclusion:**

The findings provide researchers and management of fitness Apps and women’s sports with valuable insights from the means-ends chain theory, as well as suggestions for applying App design and promotion.

## 1. Introduction

Regular exercise provides numerous benefits, including improved physical and mental health, and a reduced risk of chronic diseases. The WHO recommends that healthy adults aged 18 to 64 engage in at least 150 minutes of moderate-intensity or 75 minutes of vigorous-intensity physical activity (PA) per week.^[1]^ Despite these benefits, many individuals report barriers to maintaining regular PA, such as a lack of time, confidence, financial resources, and a supportive physical or social environment.^[2,3]^ Globally, 28% of adults aged 18 and over are physically inactive (23% of males and 32% of females), with adult females being 9% more inactive than males.^[1]^ Recent studies have shown that between 2001 and 2016, the prevalence of physical inactivity declined by 2.5% among males globally, while there was no significant change in females, resulting in greater gender disparities.^[4]^ This suggests that adult females face greater barriers to PA participation compared to adult males. In East Asia, culturally, females are more likely to assume caregiver roles in the household in addition to work, leading to less time for exercise, which adversely impacts their health.^[5]^

Fitness apps offer a platform for people to participate in PA anytime and anywhere, providing significant convenience, especially for women who face more challenges in maintaining regular PA. A survey of Chinese app users found that women accounted for 46.22% of all app users; however, among fitness app users, women comprised 56.25%.^[6]^ A study conducted in the UK revealed that women exhibited higher usage rates of fitness apps and participated in or logged longer durations of daily exercise through these apps.^[7]^ Similar findings have been confirmed in studies conducted in Saudi Arabia, Germany, and Malaysia.^[8–10]^ Despite the widespread adoption of fitness apps in recent years, user engagement tends to be short-term.^[11]^ Specifically, 74% of fitness app users stop using the app within 10 uses, and 26% use it only once after downloading.^[12]^

Recently, researchers have increasingly focused on understanding the persistence of app usage after the initial download.^[13,14]^ Many highlight the importance of user segmentation in predicting continuance intention, examining the behavior of groups such as college students,^[15]^ older adults,^[16]^ and obese individuals.^[17]^ Segmentation based on socio-demographics is a commonly used tool in marketing, helping to understand the purpose of information retrieval and its impact on both general online activity and health-specific engagement.^[18]^ Gender, one of the most researched personal characteristics, plays a key role in behavioral intentions. Identifying differences between males and females in their usage behavior will provide insights and contribute to personalized marketing strategies for fitness apps.^[19]^

Previous research has uncovered gender differences in factors influencing users’ continuance intention with fitness apps. Some researchers argue that gender influences not only users’ private internet habits but also their acceptance of health technologies.^[20]^ Additionally, factors like affiliation, privacy risk, and security risk have been shown to differ by gender in the context of fitness apps.^[19]^ Unfortunately, most current research focuses on gender variability in these factors but does not delve deeper into the usage habits and motivations of users from different genders. There is limited understanding of what factors drive female users’ continuance intention with fitness apps. Female users’ decisions are influenced not only by external features of the app but also by how the app helps them achieve their goals and values.^[21]^ Values are deeply ingrained in an individual’s sense of self, and users may be unaware of how their values shape their behavior or how their usage reflects these values.^[22,23]^

While some sports marketing studies have utilized values to interpret user behavior, there has been limited research on the relationship between sports product attributes and user values.^[24]^ Additionally, little research has focused on female users’ values in the context of fitness apps.^[25]^ Since personal experiences shape perceived value,^[26]^ this paper employs the Means-End Chain (MEC) theory to explore the experiences of female fitness app users. The MEC theory connects product attributes, the consequences experienced by users, and the perceived value of using the app, helping to explain users’ continuance intention. MEC theory has recently gained traction in sports research to identify potential mechanisms that affect individual behavior.^[24]^ This study uses MEC theory to explore the attributes, consequences, and values that contribute to female fitness app users’ continuance intention.

## 2. Theoretical background

### 2.1. The means-end chain

The MEC is a value-based cognitive model that contributes to a better understanding of decision-making and consumer behavior.^[23,27]^ It links the tangible attributes of a product (means) with highly abstract and intangible personal and emotional values (ends).^[23]^ The connotation of MEC is mainly that consumers are guided by their personal values to categorize products into various attributes that are differentiated. Consumers evaluate the main attributes of a product and make consumption choices, i.e., they choose what products to buy or not to buy, how much to buy, where to buy, and other behaviors, these behaviors are the consequences. Consumers can realize consumption value through the consequences. Product attributes are viewed as a means to an end. The “end” can reflect value tendencies and produce beneficial consequences through product attributes. Therefore, the MEC is a triple linkage system of attributes-consequences-values (ACV), in which product attributes lead consumers to produce consumption consequences and consumption consequences realize customer value.^[23,28,29]^

MEC validates a fundamental assumption that consumers buy products because they provide utility to the consumer, i.e. they fulfill customer value. Customer value is the ultimate factor that influences consumer purchasing behavior.^[30]^ As consumers hold different personal values that determine the consequences of their consumption of a product, the attributes they choose to evaluate when using or purchasing a product will naturally differ between consumers with different consumer values.^[29]^ Revealing MEC is important because attributes provide only a superficial understanding of why individuals buy and use products and services.^[31]^ Understanding how people associate product attributes with their value system is key because personal values control perception, memory, and ultimately behavior.^[29]^

In sports consumer behavior related research, MEC has been successfully used in areas such as sports tourism, recreational sports activities, and sports games.^[32–34]^ Scholars have used MEC to investigate how users choose goods or services to meet their demands. For example, Sun used MEC theory to examine the factors that influence ski tourism enthusiasts’ destination choices. Their results indicate that ski resort conditions (e.g., trail quality, snow quality) are the most important destination attributes for ski enthusiasts and have the greatest impact on their experience.^[32]^ Positive emotions, self-regulation, and self-development were values valued by ski tourism enthusiasts. Alcântara investigated runners using the MEC, and they found that runners identified with street running primarily by pursuing the values of pleasure and achievement.^[33]^ Ku found that Pokémon Go users pursued social relationships through the game using MEC and soft-laddering interview method. These connections were triggered by the benefits of making new friends, maintaining existing relationships with friends and family, and various attributes, including popularity, childhood memories, game design, and augmented reality.^[34]^

### 2.2. Female physical activity and continued use of fitness Apps

The number of inactive physically women is higher than men, but surprisingly, the number of fitness App female users is nearly 60%.^[6]^ Fitness Apps embody a great potential for promoting women’s participation in PA.^[35]^ The use of fitness Apps can help female users to participate in exercise anytime and anywhere, increase the fun of exercise, and promote their adherence to PA through certain motivational goals and communities.^[36]^ Fitness Apps are considered to be an effective tool for health self-management by promoting people’s PA.^[25]^ However, if it is not used consistently, it may lead to their lack of scientific fitness guidance and a decrease in PA level.^[37]^ On the other hand, for App developers, without consistent use by female users, the economic benefits will be greatly reduced, including revenues from sources such as advertisements, in-App purchases, subscriptions, sponsorships, etc., because of their shares.

Similar to consumer behavior, fitness App users’ continued use can be seen as consumer behavior.^[38]^ Users continue to use fitness Apps because they provide utility to the user, i.e., they deliver value.^[39]^ Value is the ultimate factor that influences users’ usage behavior.^[29]^ Although the MEC theory has been widely used to explain consumer behavior,^[24]^ few previous studies have explored the relationship between fitness App attributes and users’ values to guide value-based optimization and improvement of fitness Apps, which in turn will promote more women’s continued use of fitness Apps and increased PA levels.

In addition, China has the largest number of fitness App users in the world and a high level of mobile internet penetration.^[6]^ Therefore, it is relatively easy to promote fitness Apps in China. As such, it has the potential to become a significant fitness App market,^[6]^ and it is worthwhile to study Chinese women’s perceptions of consistently using fitness Apps for exercise.

## 3. Method

### 3.1. Research design

**Figure 1.**
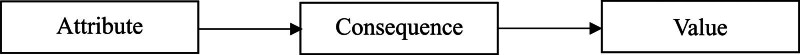
The structure of means-end chain theory (Gutman, 1982).

As shown in Figure 1, MEC is divided into 3 levels: attributes, consequences, and values. In this relationship, attributes are the tangible or visible characteristics of the product as experienced by users. The attributes help users to achieve the consequences they expect from using the product.^[23,24]^ Consequences are benefits that are directly related to the user’s use of the product.^[23]^ Users are required to interact with the attributes of the product to obtain their desired consequences.^[40]^ Value is the end state that consumers prefer as a result of using a product.^[23]^ In this research, MEC was used to determine the relationship between tangible attributes (fitness App features), the reasons why female users continue to use the fitness App (benefits/consequences sought), and the value realized through the use of the fitness App.

The laddering method is a tool commonly used by MEC to discover the factors behind user decisions.^[23]^ There are 2 types of laddering methods: hard and soft laddering. The hard laddering method obtains information mainly through questionnaires.^[23]^ The questionnaire contains hierarchical questions about attributes, consequences, and values. The hard laddering method is more appropriate for those areas of study that are well-developed.^[41]^ The soft laddering method obtains survey information primarily through qualitative, semi-structured interviews. This interview method allows respondents to answer questions without restriction.^[23]^ As this research was exploratory, the soft laddering method was more appropriate. This can better understand the connection between the fitness App attributes that users consider important and the users’ value.

**Figure 2.**
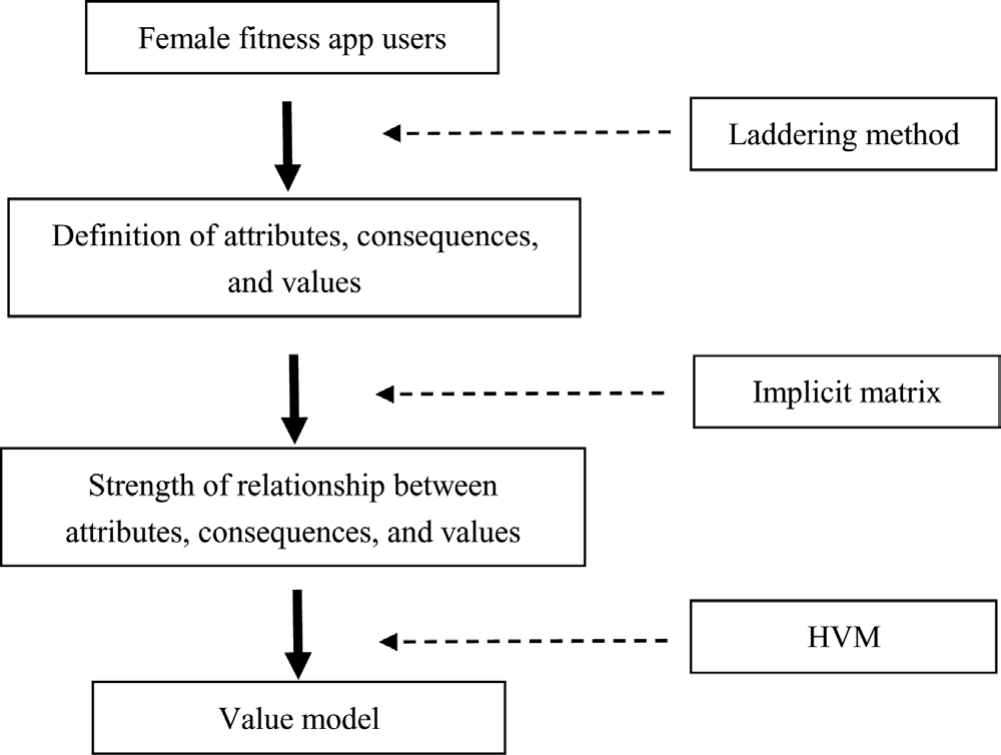
The HVM construction process for female fitness app users. HVM = hierarchical value map.

The soft laddering method was used to construct the MEC association model through the following 3 steps:

(1) Conducting one-on-one in-depth interviews: The soft laddering interview technique was employed in an unstructured format to encourage respondents to express their true feelings and thoughts about their continued use of fitness Apps in a relaxed and pleasant atmosphere. The soft laddering interview procedure was divided into 2 phases. First, interviewees were asked to explain what they liked about fitness apps. Then, a series of probing questions were used to encourage interviewees to reflect on why specific features or attributes were important to them, what consequences or benefits those features provided, and what value they believed could be derived from these benefits. The interview continued until the interviewee was unable to provide further information.
(2) Processing the information: The interview data was analyzed to extract valid insights. The content was then coded according to the attributes, consequences, and values that influenced female users’ continued use of the fitness app.
(3) Creating hierarchical value map (HVM): The ACV linkages identified in the interviews were transformed into an HVM, which illustrated the key relational paths of female users who continued using fitness apps.^[23]^ The HVM construction process for female fitness app users as shown in Figure 2.

### 3.2. Participants

Adult female users who had been consistently using the fitness Apps for at least 1 year were invited to participate in this study. Sampling criteria based on frequency of exercise required participants to exercise at least 5 times a week for at least 20 minutes using the fitness Apps. All participants volunteered to be invited for one-on-one interviews. Before beginning the interview, participants were provided with an informed consent form to explain the risks, benefits, and alternatives to the interview procedure. Participants were asked to sign if they were fully aware of their rights during the interview process and volunteered to participate in the study. Respondents then followed the interview guide to explore the fitness App user experience. A tape recorder recorded the interviews.

The interviews were conducted in May 2023 in Guangzhou, China. According to Gutman, soft laddering interviews targeting a group suggest interviewing a total of 20 to 25 people.^[23]^ For this study, 25 women were recruited through a purposive sampling method. We defined female users of fitness apps as women who use fitness apps to assist with PA. The purpose of this study was to explore the relationship between attributes, consequences, and values that influence female users’ continued use of fitness apps. It is recommended that user demographics are not separate variables because sample randomization can help to control for differences between participants.^[27]^ Interviewees ranged in age from 18 to 35. Users in this age group are the main group for fitness Apps. Data from a survey of Chinese fitness App users showed that users over the age of 35 accounted for only 27.14% of all users, while users under the age of 35 accounted for 72.86% of all users.^[6]^ The interview time for each respondent was 20-30 min, and the post-compilation manuscript was about 120,000 words. By the time the interview reached the 18th respondent, there was saturation of the resulting attributes, consequences, and values, and no new information emerged. The methodological rigor of each study was evaluated based on the SRQR (*Standards for Reporting Qualitative Research*) Guide criteria.

### 3.3. Ethics considerations

Before the interviews, the researcher thoroughly explained the purpose of the study and the interview procedures to each participant. The researcher clearly stated the rights of the respondents, including the right to refuse to answer, to end the interview, to withdraw from the study, and to have the researcher conceal any identifying information they may have provided. All respondents gave their consent and signed an informed consent form.

### 3.4. Data analysis

The analysis of the laddering interview data followed the 3 steps proposed by Gutman.^[23]^ The first was content analysis in which the interview data were coded into the attributes, consequences, and values. The second was the construction of the implication matrix, which contained the number of times each attribute was directly connected to a consequence, and each consequence had a value. The implication matrix is the most important part of MEC research because it translates the qualitative findings of content analysis into a quantitative representation of the MEC relationship.^[24]^ Finally, the HVM is generated, which visually depicts the MEC network and is the main output of the laddering analysis. A cutoff point setting between 3 and 5 is recommended in the literature before constructing an HVM.^[27]^ In this study, a cutoff value of 5 was applied to avoid losing too much information and making the HVM too complex.^[27]^

## 4. Results

### 4.1. Coding results

20 variables were extracted from the content analysis of the interviews with 25 participants, which summarized concepts with similar meanings. These variables included 9 attributes, 6 consequences, and 5 values (Table 1). In terms of frequency, the frequently mentioned attributes were instructiveness (24 times), personalization (21 times), and interaction (20 times). The consequences mentioned a lot included exercise adherence (23 times), entertainment (19 times), and peer support (18 times). As for values, what the interviews paid more attention to were health (22 times), enjoyment of life (18 times), and sense of achievement (16 times).

**Table 1:** MEC elements obtained for female users using fitness Apps.

Attribute (A)	Consequence (C)	Value (V)
Instructive	Exercise adherence	Enjoyment of life
Personalize	Entertainment	Self-fulfillment
Interactive	Peer support	Sense of achievement
Ease of use	Keep fit	Health
Convenience	High quality-price ratio	Sense of belonging
Low cost	Relaxation	
Content richness		
Good design		
Fashion		

MEC = Means-End Chain.

### 4.2. Association implication matrix

An ACV association matrix based on the number of links among attributes, consequences, and values was constructed to identify the interconnections between the elements of each tier. In terms of the frequency of attributes-consequences associations, “instructiveness-exercise adherence” (16 times) had the strongest association, followed by “interaction - entertainment” (13 times), and “personalization - exercise adherence” (12 times). In the consequences-value association analysis, exercise adherence-health (14 times) had the strongest association, followed by exercise adherence-sense of achievement (12 times) and entertainment-enjoyment of life (10 times). In order to avoid adding complexity to the HVM, the cutoff point was set to 5 in this study so as not to show the rarely mentioned ladder. The implication matrix is shown in Table 2.

**Table 2:** Implication matrix.

Attribute/consequence	Consequences						Values				
	C1	C2	C3	C4	C5	C6	V1	V2	V3	V4	V5
A1	16	9	4	3	1			2	2	4	
A2	12	4		4						3	
A3	7		11		3		3				
A4	8		4								2
A5	1	6			4					1	
A6	2				3						
A7	2					4				1	
A8	1	3	4				3				3
A9		3				2			1		
C1							4	3	12	14	3
C2							10		4		
C3							7	6	2		4
C4								2		4	
C5										3	
C6							3			4	

A1 = instructive; A2 = personalize; A3 = interactive; A4 = ease of use; A5 = convenience; A6 = low cost; A7 = content richness; A8 = good design; A9 = fashion; C1 = exercise adherence; C2 = entertainment; C3 = peer support; C4 = keep fit; C5 = high quality-price ratio; C6 = relaxation; V1 = enjoyment of life; V2 = self-fulfillment; V3 = sense of achievement; V4 = health; V5 = sense of belonging.

### 4.3. Hierarchical value map

**Figure 3.**
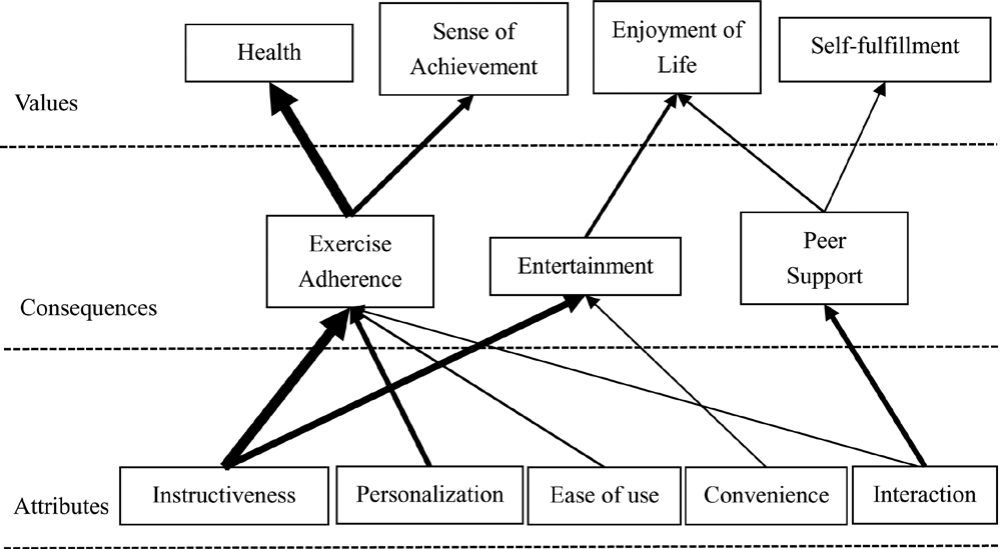
Hierarchical value map.

Based on the ACV implication matrix described above, the HVM of influencing the continuance intention of female fitness App users was plotted by representing the linking relationships between the elements through connecting lines (Fig. 3). The results suggested that the specific links that motivated participants’ continuance intention included the fitness Apps’ attributes of instructiveness, personalization, interaction, ease of use and convenience, as well as 3 main consequences: exercise adherence, entertainment and peer support, which resulted in 4 main values: enjoyment of life, sense of achievement, self-fulfillment and health.

## 5. Discussion

In this study, we constructed an ACV association matrix based on the number of links between attributes, consequences, and values influencing the continuance intention of female fitness App users, which provides a basis for the next step of drawing the HVM. The ACV matrix identifies the interconnections between the elements of each tier, with larger numbers in the ACV matrix indicating a higher number of connections and a stronger relationship.^[24]^ This concept elucidates the behavior of female users who consistently use the fitness App, emphasizing that value is created when users can correlate the product’s attributes with positive consequences to achieve their desired goals.^[27]^ As shown in the results, the higher the frequency of association, the more important the association.^[23]^

The HVM was able to visualize how female users’ perceptions of the various attributes of the fitness App were linked to explicit consequences and how these consequences helped them to achieve personal value. The thickness of the lines in the HVM represents the number of direct links between the connecting elements; the thicker the line, the more often the participant mentions these elements.^[23]^ The topmost element in the HVM is the value layer of the MEC of influencing the continuance intention of female fitness App users, the middle is the consequence layer, and the bottom is the attribute layer.

### 5.1. Instructiveness/personalization-exercise adherence-health

PA is closely related to health, and insufficient PA may lead to a variety of diseases.^[42]^ The benefits of PA usually need to be sustained over a longer period to show. However, adhering to exercise is not easy and it is easy to give up midway.^[43]^ The fitness Apps are considered to be an effective tool that can promote adherence to PA participation.^[25]^ It aims to provide users with PA guidance in various ways and means to promote active participation in PA.

“Instructiveness” plays a crucial role in the experience of fitness App users.^[44]^ Users seek professional fitness advice to ensure the effectiveness and safety of their exercise programs. Inadequate guidance may lead to program abandonment, as unscientific exercise routines fail to yield desired results and may even cause discomfort or injury, discouraging continued use. Conversely, scientifically designed exercise regimens can prevent injuries and optimize workout outcomes. Fitness apps employ various content formats, such as videos, animations, pictures, and text, to educate users on proper workout techniques, fulfilling their PA desires and needs. Many participants stressed the significance of fitness guidance. Participant #7 acknowledged the app’s meaningful guidance, remarking that “before, she and her friends paid little attention to sports and were less knowledgeable, but the fitness app’s guidance proved valuable.” Participant #11 shared a similar sentiment, highlighting how the app’s guidance alleviated post-running soreness through pre-run warm-ups, real-time monitoring, and post-run recovery exercises.

The role of “personalization” in exercise is increasingly being emphasized. Scientific fitness emphasizes that PA should be tailored to the individual and arranged and planned according to the individual’s physical condition, fitness level, etc.^[45]^ Finding the right fitness method according to one’s condition can maximize the fitness effect. Fitness apps can provide users with more targeted fitness guidance, such as personalized nutritional matching, workout schedules, and course recommendations, according to each user’s BMI, age, body shape, and other indicators, to meet the health needs of different users.^[46]^ Compared to men, women go through many special periods in their lives, such as pregnancy and menstruation. In Chinese tradition, it is believed that it is not suitable for women to participate in PA during these special stages. In fact, studies have shown that women’s participation in appropriate PA during these special times is beneficial to their health.^[47,48]^ For example, participant #13 mentioned, “Through Keep, I realized that fat loss for college girls and fat loss for mothers are 2 completely different things.” She started using the fitness app for PA more than 1 month after she gave birth, which made her body shape recover quickly, and she felt that the fitness app was especially helpful to her. This provided the motivation she needed to keep working out. In a recent study, Li found that consistent use of the fitness app helped users achieve their health goals, largely due to the personalized training plans that the fitness app tailored to the user.^[49]^ As Participant #14 said, “I use the customized exercise plan for me by fitness apps, I feel energetic every day and I would like to stick to it.”

Thus, the “personalization” and “instructiveness” attributes of fitness apps, as depicted in the HVM, correlate with users’ exercise adherence, contributing to their health goals.^[49]^

### 5.2. Interaction – peer support – enjoyment of life/self-fulfillment

As depicted in the HVM, the interaction attribute and peer support within fitness Apps are interconnected, further corroborating the significance of enjoyment of life and self-fulfillment values. Numerous studies indicate that women exhibit a greater emotional need for interaction than men, and such interaction proves more beneficial for women.^[19]^ Fitness apps offer a platform for users to communicate during exercise sessions, fostering support and encouragement among female users. Many women find this aspect integral to their enjoyment of exercising with the app. Participant #2 expressed: “My favorite aspect is interacting with fellow ‘sisters’ during Pamela’s workouts through pop-up messages. When I first began, I often struggled to find my rhythm, but whenever I sought help through pop-ups, a fellow ‘sister’ promptly responded. The support of my workout ‘sisters’ makes me feel connected and enhances my enjoyment of the experience.”

Some participants extend their online interactions offline, forging deep connections beyond the virtual realm. Participant #6 shared: “I met a wonderful ‘sister’ through the fitness app, and we became close friends, often meeting for meals and movies. We also exercise together using the app, comparing our daily workout achievements and supporting each other’s progress, it’s truly rewarding.”

In recent years, the slogan “Girls help girls” has gained global traction on social media platforms, advocating mutual support among women. While this slogan has garnered less attention in China’s mainstream media, this study unveils its impact in the realm of sports and fitness, offering fresh avenues for feminist research.

Self-fulfillment represents a higher-level pursuit. Peer support, as revealed in this study, facilitates the realization of self-fulfillment values among female users. On one hand, many women redefine beauty standards through fitness app content. Participant 11 remarked: “The traditional Chinese beauty standard dictates paleness, thinness, and youthfulness. But discussions within fitness apps often focus on showcasing toned and symmetrical bodies, which I believe epitomizes true beauty.” As women embrace autonomy and authenticity in their perception of physical beauty, their focus on body shaping becomes more aligned with self-pleasure and health considerations. On the other hand, female users derive self-fulfillment from providing help and support to their peers.

### 5.3. Instructiveness/convenience – entertainment – enjoyment of life

According to the HVM, “instructiveness” and “convenience” are linked to entertainment and the enjoyment of life. Fitness apps offer female users a platform for exercising anytime and anywhere. Users can select content that suits their schedule and interests, eliminating the need to visit a gym or attend fitness classes. This flexibility enhances the exercise experience, enabling female users to enjoy working out more easily. Participant #3 highlighted this, stating, “I often use the fitness app to meditate and relax during my lunch break at work. It helps me reset my body and mind, providing a wonderful way to unwind.”

Recent studies emphasize the impact of boredom on women’s exercise behavior.^[50]^ If women feel bored, they may be more likely to give up regular exercise.^[51]^ Enjoyment is a significant predictor of women’s participation in PA.^[50]^ Hence, creating a less monotonous exercise environment is crucial. Participants in this study reported feeling immersed in the courses and guidance provided by fitness apps, enhancing their overall enjoyment of exercise. Participant #10 explained, “Using a fitness app to exercise, I feel like time flies.” Participant #12 echoed this sentiment, saying, “I feel so happy exercising with a fitness app every day.” Therefore, the instructive features of fitness apps contribute to a more entertaining exercise environment, making workouts less dull and encouraging users to remain committed.

Moreover, if exercise demands high self-discipline, it might become tedious for some individuals, leading to dropout.^[50,52]^ The convenience offered by fitness apps provides female users with more choices, reducing the need for strict self-control and allowing them to relax during workouts, thus enhancing the experience.

The benefits of convenience extend to privacy during workouts. For some female users, fitness is a private activity, and they prefer not to be observed or discussed. Participant #4 shared, “I usually work out in my living room using the fitness app. I don’t have to worry about being approached, and I can focus entirely on my workout. I love this feeling.” Previous studies have shown that individuals can better enjoy an experience when their privacy is protected.^[53]^ If privacy concerns persist, individuals may experience tension or anxiety, hindering their focus.^[54]^ Shyness and fear of ridicule have been significant factors limiting women’s participation in PA.^[55]^ Traditional gender-related views have objectively hindered some women from engaging in sports activities.

The relationship between entertainment and enjoyment of life has been established in previous studies.^[50]^ Therefore, creating a relaxed and enjoyable exercise environment is crucial for women’s fitness. Participant #4 noted, “I prefer to exercise at home in the evening with my favorite music playing. It’s cozy and comfortable, the best time for me after a long day at work.” This study found that convenience influences women’s enjoyment during workouts, thereby promoting their participation in PA. One significant advantage of fitness apps is their ability to provide users with a platform for exercise anytime, anywhere. Female users can work out in environments where they feel relaxed and uninterrupted, enabling them to fully enjoy the pleasure of exercise and appreciate the beauty of life.

### 5.4. Interaction/ease of use – exercise adherence – sense of achievement

Interaction features not only foster peer support but also significantly impact users’ exercise adherence. Fitness platforms employ various interaction methods and incentives to encourage users to maintain their workout routines. For instance, many fitness apps prominently display users’ exercise duration and streaks on the home page, utilizing virtual badges and tangible rewards to motivate continued engagement. Additionally, hosting diverse challenges serves to retain users’ interest and commitment to exercise. Furthermore, user-to-user interactions, such as sharing photos, check-ins, and liking posts, effectively motivate individuals to remain consistent with their workouts.^[44]^

Ease of use is paramount to the user experience, particularly for female fitness app users.^[56]^ A user-friendly interface that facilitates easy navigation is essential for accessing the app’s features and content seamlessly.^[57]^ This includes streamlined processes like registration, login, goal setting, and content access. When female users perceive a fitness app as easy to use, they are more inclined to continue using it and adhere to their workout routines.^[56]^ For example, Participant #2 remarked, “Keep’s interface is straightforward; I can easily find the courses I want on the home page. Unlike some other apps, where searching is a hassle.” Participant #18 echoed this sentiment, stating, “Keep’s ease of use keeps me engaged.” Conversely, if an app is not user-friendly, users may feel frustrated and deterred from continued usage.

While past studies suggested that ease of use primarily influences the initial adoption of information systems, its significance extends to sustained app usage.^[58]^ In this study, ease of use was found to significantly impact users’ continued use of fitness apps. Participant #11 highlighted instances where updates made apps more memory-intensive, leading to difficulties in finding desired information despite improved functionality and interface. Participant #6 remarked, “I avoid updating because the current version is so convenient; I can access the desired course with a single click.” Such issues can profoundly affect the user experience and potentially lead to app abandonment.

Female fitness app users often derive a sense of achievement from reaching small milestones. By consistently utilizing fitness apps for exercise, female users can monitor their progress and assess goal attainment, such as weight loss, muscle gain, or endurance improvement. Tangible outcomes visible on the fitness app provide female users with a sense of achievement. Participant #14 explained, “Reviewing my weight records on the fitness app brings me a great sense of achievement.” Witnessing the results of their efforts boosts female users’ confidence and motivates them to persist with fitness app usage, striving for further goals.

In summary, fitness Apps have shown great potential for promoting PA in women.^[25]^ Although a higher percentage of women than men are physically inactive, the number of female users of fitness Apps accounts for nearly 60% of the population.^[6]^ Fitness Apps provide a comprehensive solution for women, encouraging them to be more physically active and achieve lasting success. This trend will continue to lead women towards healthier, more active lifestyles. The impact of the Covid-19 pandemic is another major milestone in this direction.^[59]^

According to MEC theory, users are “goal-oriented decision makers who choose to perform behaviors that seem most likely to lead to desired outcomes.”^[23,27]^ This study used HVMs constructed through MEC and soft laddering interview techniques to explore fitness App users’ highly abstract and intangible values and to discover tangible attributes that were important to female users, and the consequences of their experience of using them.

Our findings from the target population may be beneficial for the sustainability of fitness apps to promote more female users to use fitness apps for continued PA. The results of the study indicated that participants perceived instructiveness, personalization, interaction, convenience, and ease of use as the most important attributes of fitness Apps; the consequences were peer support, entertainment, and exercise adherence; and the values for female users were enjoyment of life, sense of achievement, self-fulfillment, and health.

In addition, the research findings provide some theoretical and practical contributions, which are discussed below.

### 5.5. Theoretical contributions

This paper contributes to the existing research on fitness app user behavior. Firstly, this is the first MEC research in the literature to explore female fitness app user behavior after the end of the Covid-19 pandemic. The findings contribute to the promotion of female users’ continued use of fitness Apps for PA. Secondly, attributes such as personalization, instructiveness, interaction, convenience, and ease of use are features offered by fitness Apps that are valued by female users. In the information systems success model, the characteristics of successful systems include information quality, service quality, and system quality.^[60]^ The instructiveness attribute in this research was similar to the model in terms of information quality, the personalization attribute was similar to service quality, and the ease of use attribute was comparable to system quality. Unfortunately, the information systems success model did not consider the constructs of interaction and convenience, which are crucial for female fitness App users. Therefore, future scholars might consider the constructs of interaction and convenience.

### 5.6. Practical impact

Increasing competition in the fitness App sector has intensified the need for businesses to constantly optimize and innovate in order to achieve a competitive edge. Female users deserve extra attention as a major user group of fitness Apps. In this research, the 5 key attributes valued by female users are: instructiveness, convenience, ease of use, personalization and interaction. This research found 3 consequences: peer support, entertainment, and exercise adherence. The consequence of peer support stems from the feature of interaction, the consequence of entertainment stems from the feature of instructiveness and convenience, and the consequence of exercise adherence stems from the feature of instructiveness, interaction, personalization, and ease of use. Therefore, when developing interaction features, developers need to consider if the feature may help users obtain peer support; when designing convenience-related features, they should consider whether they can provide an entertaining experience for female users. In addition, when designing features of instructiveness and personalization, designers must consider whether it will help female users to keep exercising.

In this study, 4 values are presented that are valued by female users who consistently use fitness apps: enjoyment of life, health, self-fulfillment, and sense of achievement. These values can provide operators with advertising factors, as female users tend to invest money and time in this area. Therefore, this research provides essential insights and significant implications for scholars, developers, managers, or operators who are interested in this research or in using fitness technology.

### 5.7. Limitations and future research

This paper provided some insights. However, it also had some limitations. On the one hand, the research identified some key connections between attributes, consequences, and values valued by female fitness app users through qualitative methods, but it was difficult to conduct the survey on a large scale due to the limitations of the number of interviews. It is recommended that future studies try to obtain data by means of a large-scale questionnaire survey to confirm the results by quantitative methods. On the other hand, due to the differences between the cultures of different countries, the study may not necessarily be applicable to female fitness app users in other cultures, and future research could investigate users in different countries to further improve the study of female fitness app user behavior.

## 6. Conclusion

We can find from the result that different female users use fitness Apps for different purposes. Although fitness apps are recognized as an effective way to promote PA in women, only sustained use can yield benefits.^[61]^ This paper extended the MEC epistemological status and explores the layered character of female users’ sustained use of fitness apps. It is believed that when individuals actually like what they are doing, intrinsic motivation is longer-lasting and more powerful.^[62]^ It is currently uncertain how much of an impact the use of fitness Apps has on women’s personal exercise.^[63]^ However, in the long run, fitness Apps can help to increase the enjoyment of life, sense of achievement, self-fulfillment, and health value of exercise for female users by providing incentives such as instructiveness, personalization, interaction, convenience, and ease of use. Therefore, it is crucial to improve or provide ongoing incentives to encourage women to participate in PA sustainably.

## Author contributions

**Conceptualization:** Le Lyu, Nor Eeza Zainal Abidin.

**Data curation:** Hutkemri Zulnaidi, Nor Eeza Zainal Abidin.

**Validation:** Hutkemri Zulnaidi.

**Writing – original draft:** Le Lyu, Nor Eeza Zainal Abidin.

**Writing – review & editing:** Hutkemri Zulnaidi.
